# Correspondence to: Estimating the full health and economic benefits of current and future influenza vaccines

**DOI:** 10.1186/s12916-023-02996-3

**Published:** 2023-08-09

**Authors:** Naomi R. Waterlow, Simon R. Procter, Rosalind M. Eggo, Mark Jit

**Affiliations:** Centre for Mathematical Modelling of Infectious Disease, London School of Hygiene and Tropical Medicine, London, UK

**Keywords:** Influenza, Vaccines, Next-generation vaccines, Cost-effectiveness, Burden, Mathematical modelling, Complexity, Assumptions

## Abstract

We recently published an article in *BMC Medicine* looking at the potential health and economic impact of paediatric vaccination using next-generation influenza vaccines in Kenya: a modelling study. In their commentary on our article, Lafond et al. highlight the potential importance of the wider benefits of vaccination on cost-effectiveness. Whilst we agree with many points raised in the commentary, we think it raises further interesting discussion points, specifically around model complexity, model assumptions and data availability. These points are both relevant to this manuscript but have wider implications for vaccine cost-effectiveness studies.

## Commentary

We thank Lafond et al. for their commentary on our cost-effectiveness analysis of next-generation influenza vaccines [1]. Lafond et al. raise some important points, highlighting the conservative nature of our estimates of the cost-effectiveness of influenza vaccines. Our study was limited by the data available, and we therefore agree we could not include some wider benefits that would potentially increase the economic impacts of both current and next-generation influenza vaccines.

Lafond et al.’s commentary raises some further discussion points, firstly, the complex and important role of assumptions in the modelling work. For example, they discuss that vaccine effectiveness assumptions made may not capture the full benefits, as we do not consider the disease-modifying effects of vaccination but instead assume that vaccine immunity is sterilising (i.e. vaccinees are either fully protected against infection or not protected at all). Whilst this may result in underestimating benefits if vaccines are also highly effective against disease, it may also result in an overestimate of the benefits, because sterilising immunity in the model gives both direct protection of the individual and indirect protection to others—you cannot transmit influenza if you do not get infected. If the vaccine is instead only disease-modifying, transmission will still occur, potentially increasing the number of cases while decreasing the chance of severe disease. This demonstrates that changing assumptions has complex effects, and in our analysis, we aimed for an appropriate balance whilst acknowledging the limitations of such assumptions.

The second area of discussion raised is the inclusion of broader benefits of vaccination, such as the impact on antimicrobial resistance (AMR). Lafond et al. rightly highlight the importance of these benefits, and that in some circumstances it can be viable to quantify them. For our study however, local information needed for this, for example on age-specific antimicrobial prescribing for influenza, was not available. AMR rates vary enormously by location, both nationally and sub-nationally [2] and therefore using information from further afield in our Kenya analysis could be inappropriate. As Lafond et al. note, even wider impacts such as health equity could also have been included, but are beyond the scope of this (and many other) cost-effectiveness studies.

We agree with Lafond et al. that it is important to raise awareness of the wider benefits of influenza vaccines that may underpin their full economic value [3, 4] and, where they cannot be easily quantified, to weigh them qualitatively in any decision. Finally, we note that the inclusion of such additional benefits of influenza vaccination would reinforce our main conclusion that next-generation vaccines could be a cost-effective intervention in Kenya.

## Data Availability

Not applicable.

## Abbreviation

AMR: Antimicrobial resistance

## References

[1] Waterlow NR, Radhakrishnan S, Dawa J, van Leeuwen E, Procter SR, Lambach P (2023). Potential health and economic impact of paediatric vaccination using next-generation influenza vaccines in Kenya: a modelling study. BMC Med.

[2] Global antimicrobial resistance and use surveillance system (GLASS) report: 2022. Available: https://www.who.int/publications-detail-redirect/9789240062702.

[3] Hutubessy RCW, Lauer JA, Giersing B, Sim SY, Jit M, Kaslow D, et al. The Full Value of Vaccine Assessments (FVVA): a framework to assess and communicate the value of vaccines for investment and introduction decision making. BMC Med. 2023. 10.1186/s12916-023-02929-0 PMC10318807 37400797

[4] Gessner BD, Kaslow D, Louis J, Neuzil K, O’Brien KL, Picot V (2017). Estimating the full public health value of vaccination. Vaccine.

